# Small Heterodimer Partner Modulates Macrophage Differentiation during Innate Immune Response through the Regulation of Peroxisome Proliferator Activated Receptor Gamma, Mitogen-Activated Protein Kinase, and Nuclear Factor Kappa B Pathways

**DOI:** 10.3390/biomedicines11092403

**Published:** 2023-08-28

**Authors:** Forkan Ahamed, Natalie Eppler, Elizabeth Jones, Lily He, Yuxia Zhang

**Affiliations:** Department of Pharmacology, Toxicology and Therapeutics, University of Kansas Medical Center, MS 1018, 3901 Rainbow Boulevard, Kansas City, KS 66160, USA; fahamed@kumc.edu (F.A.); neppler2@kumc.edu (N.E.); ejones11@kumc.edu (E.J.); lhe@kumc.edu (L.H.)

**Keywords:** nuclear receptor, small heterodimer partner (SHP), knockout, macrophage, differentiation

## Abstract

Hepatic macrophages act as the liver’s first line of defense against injury. Their differentiation into proinflammatory or anti-inflammatory subpopulations is a critical event that maintains a delicate balance between liver injury and repair. In our investigation, we explored the influence of the small heterodimer partner (SHP), a nuclear receptor primarily associated with metabolism, on macrophage differentiation during the innate immune response. During macrophage differentiation, we observed significant alterations in *Shp* mRNA expression. Deletion of *Shp* promoted M1 differentiation while interfering with M2 polarization. Conversely, overexpression of SHP resulted in increased expression of peroxisome proliferator activated receptor gamma (*Pparg*), a master regulator of anti-inflammatory macrophage differentiation, thereby inhibiting M1 differentiation. Upon lipopolysaccharide (LPS) injection, there was a notable increase in the proinflammatory M1-like macrophages, accompanied by exacerbated infiltration of monocyte-derived macrophages (MDMs) into the livers of *Shp* myeloid cell specific knockout (*Shp*-MKO). Concurrently, we observed significant induction of tumor necrosis factor alpha (*Tnfa*) and chemokine (C-C motif) ligand 2 (*Ccl2*) expression in LPS-treated *Shp*-MKO livers. Additionally, the mitogen-activated protein kinase (MAPK) and nuclear factor kappa B (NF-κB) pathways were activated in LPS-treated *Shp*-MKO livers. Consistently, both pathways were hindered in SHP overexpression macrophages. Finally, we demonstrated that SHP interacts with p65, thereby influencing macrophage immune repones. In summary, our study uncovered a previously unrecognized role of SHP in promoting anti-inflammatory macrophage differentiation during the innate immune response. This was achieved by SHP acting as a regulator for the Pparg, MAPK, and NF-κB pathways.

## 1. Introduction

The liver serves as a primary target for the innate immune response due to its continuous exposure to microorganisms and products originating from the gut [1,2]. Within the liver, monocytes/macrophages, along with granulocytes and dendritic cells, act as key effector cells of the innate immune system. Hepatic macrophages are primarily composed of two distinct types: resident macrophages known as Kupffer cells (KCs), which originate from erythromyeloid progenitors derived from the yolk sac; and monocyte-derived macrophages (MDM). In the context of inflammation, monocytes migrate from the peripheral circulation to the liver, where they differentiate into tissue macrophages. These macrophages play crucial roles in functions such as phagocytosis of foreign particles and cellular debris, antigen presentation to lymphocytes, secretion of cytokines, modulation of immune responses, and the restoration of a normal tissue environment [3,4].

Macrophages possess remarkable plasticity, allowing them to adapt their phenotypes and functions in response to environmental cues. In vitro studies have revealed that macrophages can be broadly categorized into two major populations based on their distinct phenotypes: classically activated proinflammatory M1 macrophages and alternatively activated anti-inflammatory M2 macrophages [5,6]. However, it is worth noting that certain macrophages, such as tumor-associated macrophages, may exhibit characteristics that overlap between these two groups [7]. The prototypical signals triggering M1 proinflammatory activation include interferon gamma (IFN-gamma), lipopolysaccharide (LPS), and tumor necrosis factor alpha (TNFα), while interleukin 4 (IL4) serves as a key signal for anti-inflammatory M2 activation. Although the M1/M2 classification may oversimplify the intricate biological response of macrophages in vivo, numerous studies have substantiated that macrophage differentiation into distinct proinflammatory or anti-inflammatory phenotypes significantly influences host defense and the pathogenesis of various liver diseases [8,9,10]. Therefore, the identification of heterogeneous macrophage populations and a comprehensive understanding of the molecular mechanisms governing macrophage heterogeneity are crucial for assessing disease progression, evaluating treatment outcomes, and developing targeted therapeutics that specifically modulate macrophage function [11,12,13,14,15].

Our study aimed to investigate the involvement of small heterodimer partner (Nr0b2, *Homo sapiens SHP*; *Mus musculus Shp*) in macrophage differentiation during the innate immune response. SHP is a nuclear receptor lacking a DNA binding domain and known endogenous ligands [16]. It functions as a negative regulator of gene transcription and plays a crucial role in the regulation of bile acid, glucose, and energy metabolism through its interactions with other nuclear receptors and transcription factors [16,17,18,19,20]. Recent studies have shed light on a novel function of SHP in inflammation [21,22,23], where it acts as a negative regulator of immune response [24]. Mice lacking *Shp* are more susceptible to endotoxin-induced sepsis and concanavalin A-induced hepatitis [16,25,26,27], while inducing SHP expression has been found to ameliorate systemic inflammatory responses [23]. Moreover, we have recently discovered an anti-inflammatory role of SHP during the development of nonalcoholic steatohepatitis (NASH), where the loss of *Shp* in hepatocytes triggers nuclear factor kappa B (NF-κB) activation and the release of chemokine (C-C motif) ligand 2 (CCL2), exacerbating liver inflammation and fibrosis [28,29].

While the role of SHP in repressing innate immune activation has been well documented, its involvement in macrophage differentiation during the innate immune response remains unclear. To address this gap in knowledge, we utilized a cell-type-specific knockout mouse model to examine the role of SHP in macrophage differentiation. We found that *Shp* mRNA was downregulated in proinflammatory M1 macrophages, but upregulated in anti-inflammatory M2 macrophages. Deletion of *Shp* promoted M1 macrophage differentiation while interfering with M2 macrophage polarization. Conversely, overexpression of SHP resulted in reduced expression of miR-34a, leading to increased expression of peroxisome proliferator activated receptor gamma (*Pparg*) with decreased M1 differentiation. Consistently, in the *Shp* myeloid cell specific knockout (*Shp*-MKO) mouse model, we observed increased hepatic infiltration of monocytes and M1 macrophage differentiation following LPS challenge, accompanied by augmented activation of the mitogen-activated protein kinase (MAPK) and NF-κB pathways resulting from the loss of macrophage *Shp* in *Shp*-MKO. In summary, our study sheds light on the crucial role of SHP in modulating hepatic macrophage differentiation, contributing to the regulation of the inflammatory response and immune balance in the liver.

## 2. Materials and Methods

### 2.1. Cell Lines, Chemicals, Plasmids, and Antibodies

Mouse macrophage cell line RAW 264.7 cells from the American Type Culture Collection (ATCC, Manassas, VA, USA, Cat. No. TIB-71) were cultured in Corning™ Dulbecco’s Modified Eagle’s Medium (Fisher, Waltham, MA, USA, cat. MT10013CV) supplemented with Gibco™ 100 units/mL penicillin G-streptomycin sulfate (Fisher, 15-140-122) and 10% heat-inactivated fetal bovine serum (Fisher, 10-082-147). To achieve overexpression of FLAG-SHP, a lentiviral vector pMSCV-puro (Clontech, Mountain View, CA, USA, cat. 631461) was employed, and stable overexpression cells were selected using puromycin (Fisher, Waltham, MA, USA, cat. A1113802). The cells were treated with 100 ng/mL lipopolysaccharide (LPS, Sigma-Aldrich, St. Louis, MO, USA, cat. L2654) for various time intervals (0, 5, 10, 30, and 60 min) for subsequent Western blot analysis. For Western blotting, immunohistochemistry staining, and immunoprecipitation, the following antibodies were utilized: β-actin (Sigma, A-1978), phospho-JNK (Thr-183/Tyr-185) (Cell Signaling Technology, Danvers, MA, USA, cat. 4668), JNK (Cell Signaling Technology, 9252), phospho-c-Jun (Ser-63) (Cell Signaling Technology, 2361), α-tubulin (Sigma, T6074), histone H3 (Cell Signaling Technology, 14269), phosphor-TAK1 (Ser-412) (Cell Signaling Technology, 9339), TAK1 (Cell Signaling Technology, 4505), phospho-SEK1/MKK4 (Ser257) (Cell Signaling Technology, 4514), SEK1/MKK4 (Cell Signaling Technology, 9152), phospho-IKKα (Ser176)/IKKβ (Ser177) (Cell Signaling Technology, 2078), IKKβ (Cell Signaling Technology, 8943), phospho-IκBα (Ser32/36) (Cell Signaling Technology, 9246), IκBα (Cell Signaling Technology, 4814), NF-κB p65 (Cell Signaling Technology, 8242), and F4/80 (Cell Signaling Technology, 70076).

### 2.2. Animal Studies

C57BL/6J mice (stock no. 000664) were procured from the Jackson Laboratory (Bar Harbor, ME, USA). *Shp*^flox/flox^ mice, generously provided by Drs. Johan Auwerx and Kristina Schoonjans at the Ecole Polytechnique de Lausanne (Lausanne, Switzerland), were backcrossed into the C57BL/6J background for 10 generations. *Shp*^flox/flox^ mice were crossed with LysM-Cre mice (Jackson Laboratory, Stock No: 004781) to generate heterozygous mice. Subsequently, the heterozygous mice were bred to obtain *Shp* myeloid cell specific knockout (*Shp*-MKO represents *Shp*^flox/flox^; LysMcre positive) and their littermate wild-type controls (WT represents *Shp*^flox/flox^; LysMcre negative). Mice were housed in a virus-free facility with a 12 h light/dark cycle (lights on from 6 a.m. to 6 p.m.) and maintained at a temperature of 25 °C, with ad libitum access to food and water. Male mice aged 8–10 weeks were used for the experiments, unless otherwise stated (*n* = 5/group). In the LPS injection experiment, both WT and *Shp*-MKO mice received intraperitoneal injection of LPS at 1 mg/kg body weight. Samples were collected at 0-, 3-, and 7 h post-injection. For the bone marrow-derived macrophage polarization experiment, a published protocol was followed [30]. In brief, the femur and tibia were collected from the mice, and bone marrow cells were differentiated into macrophages using mouse macrophage colony-stimulating factor (M-CSF, R&D Systems™, 416ML010) at 10 ng/mL for 7 days. On the 7th day, the differentiated macrophages were cultured with IFN-gamma (100 ng/mL) or IL4 (50 ng/mL) for 24 h to induce M1 or M2 macrophage polarization, respectively. All experiments were conducted in compliance with relevant guidelines and regulations approved by the Institutional Animal Care and Use Committee (ICAUC) at the University of Kansas Medical Center.

### 2.3. Hepatic Cell Isolation and Flow Cytometry Analysis

Hepatic cell isolation and purification were conducted at the Kansas University Medical Center Cell Isolation Core, following a previously described method [31] with slight modifications. In brief, mouse livers were perfused with 25 mL of solution I (9.5 g/liter Hanks’ balanced salt solution, 0.5 mmol/liter EGTA, pH 7.2), followed by 50 mL of solution II (9.5 g/liter Hanks’ balanced salt solution, 0.14 g/liter collagenase IV, and 40 mg/liter trypsin inhibitor, pH 7.5). After digestion, a single-cell suspension was obtained and filtered through a 100 μm Falcon cell strainer (Fisher Scientific, Waltham, MA, USA, cat. 08-771-19). The cells were centrifuged at 50× *g* for 5 min at 4 °C to pellet hepatocytes. The supernatant containing nonparenchymal cells (NPCs) was then centrifuged at 300× *g* for 10 min at 4 °C to enrich NPCs. In hepatic macrophage polarization experiment, macrophages were captured from NPCs by CD11b MicroBeads (Miltenyi Biotec Inc., San Jose, CA, USA, cat. 130-049-601) and differentiated into M1 or M2 macrophages using DMEM media supplemented with IFN-gamma (100 ng/mL) or IL4 (50 ng/mL) for 24 h, respectively. In flow cytometry experiment, approximately 1 × 10^6^ NPCs were incubated with anti-mouse CD16/CD32 (TruStain FcX, BioLegend, San Diego, CA, USA, cat. 101319) diluted in FACS buffer (2 mM EDTA, 10% FBS in PBS) for 15 min on ice to block non-specific antibody binding. Subsequently, the cells were incubated with the Brilliant Violet 605™ CD45 (BioLegend, USA, 103139), Brilliant Violet 421™ CD11b (BioLegend, San Diego, CA, USA, cat. 101235), PE/Cyanine7 Ly-6C (BioLegend, USA, 128017) anti-mouse antibodies, and the fixable viability dye (Zombie Aqua, BioLegend, 423101) for 30 min on ice. After centrifugation (300× *g*) for 5 min, the cells were washed twice with 1 mL of PBS for 5 min and finally resuspended in 300 µL of FACS buffer. The cells were then analyzed using a FACS Calibur instrument (BD, Franklin Lakes, NJ, USA). FlowJo-V10 software was used for data analysis.

### 2.4. Liver Histology and Immunohistochemistry

Fresh liver tissues were fixed with 10% formalin (Fisher, SF100) to preserve their structural integrity. Paraffin sections of 5 μm thickness were prepared and subjected to staining with hematoxylin and eosin (H&E) for general tissue examination, as well as immunohistochemical staining. For the immunohistochemical staining of F4/80, the paraffin sections were rehydrated and treated with 0.3% hydrogen peroxide in PBS for 15 min to block endogenous peroxidase activity. Antigen retrieval was achieved by boiling the sections in sodium citrate buffer (pH 6.0) for 5 min using a pressure cooker. Subsequently, the slides were treated with 5% normal serum for 30 min to block non-specific binding, followed by overnight incubation with rabbit anti-mouse F4/80 antibody at 4 °C. For the final detection, an ImmPRESS peroxidase polymer detection kit (Vector Laboratories, Newark, CA, USA, cat. MP-7444) and ImmPACT 3,3′-diaminobenzidine peroxidase substrate (Vector Laboratories, SK-4105) were utilized. After thorough washing, the sections were counterstained with hematoxylin, dehydrated, cleared, and mounted. Microscopic images were captured using a BX60 microscope, and the area of positive staining for DAB (3,3′-diaminobenzidine) was quantified using ImageJ 1.53t software.

### 2.5. Real-Time Quantitative PCR

Real-time quantitative PCR (qPCR) analysis was performed using the SYBR Green PCR master mix (Applied Biosystems, Foster City, CA, USA, cat. 4309155), following the previously described protocols [28,29,32]. The specific primer sequences utilized for the qPCR are provided in Table S1. The hsa-miR-34a-5p LNA™ PCR primer set (Exiqon, Woburn, MA, USA, cat. 204486) was used to measure the expression level of miR-34a. The abundance of PCR products was quantified using threshold cycle (Ct) values, and the relative ratio of specific genes to the housekeeping gene *actin* was determined. The resulting values were then presented as the fold change in the tested group compared to the control group.

### 2.6. Western Blotting and Immunoprecipitation

Mouse liver tissues were prepared for protein analysis using the following procedures. First, the tissues were homogenized using a PowerGen 700 homogenizer (Fisher Scientific, Waltham, MA, USA) in lysis buffer containing protease inhibitors (Fisher Scientific, protease inhibitor mixture PI78410). The lysis buffer consisted of 50 mM Tris (pH 7.5), 1% Nonidet P-40, 150 mM NaCl, 0.5% sodium deoxycholate, and 0.1% SDS, ensuring efficient extraction of whole protein lysates. For the extraction of nuclear and cytoplasmic proteins, a commercial kit (Fisher, PI78833) was utilized according to the manufacturer’s instructions. Next, protein lysates (60 μg) were separated by SDS-PAGE and transferred to nitrocellulose membranes. The membranes were then blocked and incubated with primary antibodies specific to the target proteins. Subsequently, horseradish peroxidase-conjugated secondary antibodies were applied, allowing for the detection of antibody binding. The visualization of antibody-bound proteins was achieved using either SuperSignal West Pico Plus Chemiluminescent Substrate (Fisher, PI34580) or SuperSignal West Femto Chemiluminescent Substrate (Fisher, PI34094). Images were captured using an Odyssey XF LI-COR imaging system (Lincoln, NE, USA). To ensure equal protein loading, loading controls such as β-actin, α-tubulin, and histone H3 were included and verified. Quantitative analysis of band intensity was performed using Image Studio Lite 5.2 software, and the relative expression levels were normalized to the loading controls. For the immunoprecipitation experiment, 1000 μg of whole protein lysates from control PMSCV cells and PMSCV-SHP cells overexpressing FLAG-SHP were incubated with 2 μg of anti-FLAG M2 magnetic beads (Sigma-Aldrich, St. Louis, MO, USA, cat. M8823). The immune complexes were captured using a magnetic stand, and subsequent elution was performed using 2× SDS loading buffer. The pulldown of p65 and FLAG-SHP was detected by Western blotting. A TrueBlot^®^ anti-rabbit IgG HRP (Rockland, Pottstown, PA, USA, cat. RL18-8816-33) was used as a secondary antibody, as this antibody does not interfere with the immunoprecipitation of immunoglobulin heavy and light chains, ensuring accurate detection.

### 2.7. Statistical Analysis

GraphPad Prism 8.0 (GraphPad Software, La Jolla, CA, USA) was used for data analysis. The quantitative data are presented as the mean ± SEM. Statistical analysis was performed using Student’s *t*-test to determine the significant difference between two groups. For comparisons among multiple groups, one-way analysis of variance (ANOVA) was conducted, followed by Duncan’s test. Statistical significance was considered at a 95% confidence level.

## 3. Results

### 3.1. Macrophage Differentiation Alters Shp mRNA Expression

**Figure 1:**
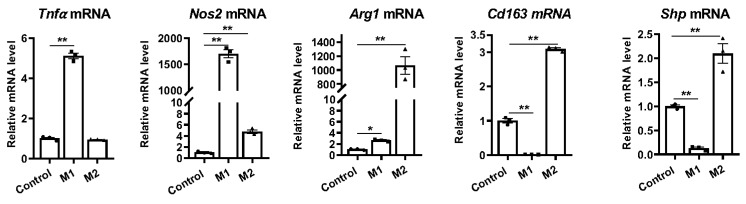
Macrophage differentiation alters *Shp* mRNA expression. The livers of C57BL/6 mice were perfused and digested to harvest nonparenchymal cells (NPCs). Hepatic macrophages were then isolated from NPCs by CD11b MicroBeads and differentiated into M1 or M2 macrophages using DMEM media supplemented with IFN-gamma (100 ng/mL) or IL4 (50 ng/mL) for 24 h, respectively. The relative mRNA levels of M1 markers (*Tnfa* and *Nos2*), M2 markers (*Arg1* and *Cd163*), and *Shp* were determined using quantitative PCR (qPCR). Data are presented as mean ± SEM for 3 samples/group. * *p* < 0.05 and ** *p* < 0.01 between the indicated groups.

To investigate the impact of macrophage differentiation on the expression of *Shp*, we conducted experiments using hepatic macrophages isolated from C57BL/6J liver. The macrophages were differentiated into either proinflammatory M1 or anti-inflammatory M2 macrophages by treating them with IFN-gamma (100 ng/mL) or IL4 (50 ng/mL), respectively. Following a twenty-four-hour incubation, successful differentiation into M1 or M2 macrophages was confirmed by observing significant upregulation of genes encoding proinflammatory markers *Tnfa* and nitric oxide synthase 2 (*Nos2*) in M1 macrophages, as well as the anti-inflammatory markers arginase 1 (*Arg1*) and CD163 antigen (*Cd163*) in M2 macrophages (Figure 1). Interestingly, we observed that M1 differentiation led to the inhibition of *Shp* mRNA expression, while M2 differentiation resulted in its increased expression (Figure 1). These findings strongly suggest that macrophage differentiation alters *Shp* mRNA expression.

### 3.2. Shp Deletion in Macrophages Enhances M1 Polarization but Impairs M2 Differentiation

**Figure 2:**
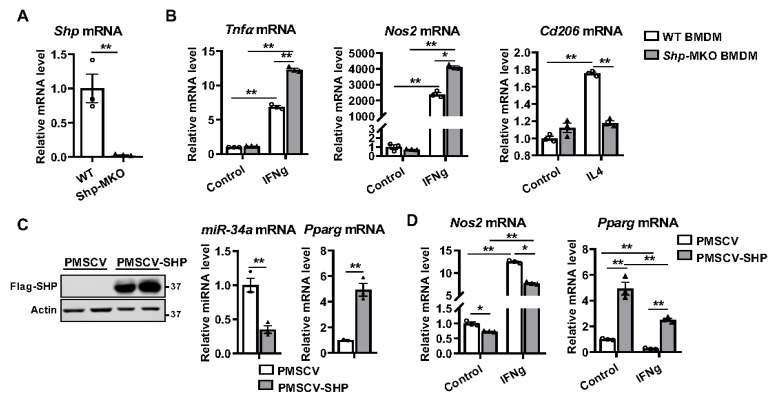
SHP regulates macrophage differentiation in vitro. (**A**) Peritoneal macrophages were isolated from WT and *Shp*-MKO mice, and the relative mRNA levels of *Shp* were determined by qPCR. (**B**) Bone marrow cells were isolated from WT and *Shp*-MKO mice and differentiated into macrophages using M-CSF (10 ng/mL) for 7 days. On the 7th day, the differentiated macrophages were cultured with IFN-gamma (100 ng/mL) or IL4 (50 ng/mL) for 24 h to induce M1 or M2 macrophage polarization, respectively. The mRNA expression of *Tnfa*, *Nos2*, and *Cd206* was determined using qPCR. (**C**) Left, Western blot confirmed the overexpression of Flag-SHP in mouse macrophage RAW cells. Right, the expression of *miR-34a* and *Pparg* was determined by qPCR. (**D**) RAW cells with or without SHP overexpression were treated with IFN-gamma (100 ng/mL) for 24 h. qPCR was employed to measure mRNA levels of *Nos2* and *Pparg*. The data are presented as mean ± SEM for 3 samples/group. * *p* < 0.05 and ** *p* < 0.01 between the indicated groups.

To assess the role of SHP in regulating macrophage differentiation, we crossed *Shp*^flox/flox^ with LysMcre mice and generated *Shp*-MKO and WT controls. Confirmation of *Shp* deletion from myeloid cells was achieved through qPCR analysis of peritoneal macrophages isolated from both the *Shp*-MKO and littermate WT controls (Figure 2A). Subsequently, we performed macrophage differentiation experiments using bone marrow cells obtained from the WT and *Shp*-MKO mice, which were cultured with macrophage colony-stimulating factor (M-CSF) for 7 days to generate bone marrow-derived macrophages (BMDMs). On the seventh day, the differentiated BMDMs were treated with either IFN-gamma or IL4. Remarkably, IFN-gamma treatment significantly increased the mRNA expression of proinflammatory M1 markers, *Tnfa* and *Nos2*, in WT macrophages (Figure 2B). Strikingly, this effect was augmented in the *Shp* knockout macrophages (Figure 2B), indicating that the absence of *Shp* enhanced M1 macrophage polarization. Conversely, M2 anti-inflammatory macrophage differentiation was impaired in the *Shp* knockout macrophages, resulting in a reduced induction of M2 marker mannose receptor C type 1 (*Mrc1* or *Cd206*) mRNA compared to the WT after IL4 treatment (Figure 2B). These findings clearly demonstrate that knocking out *Shp* in macrophages enhanced M1 polarization while impairing M2 polarization.

### 3.3. SHP Overexpression Increases the Expression of Pparg and Inhibits M1 Differentiation

To investigate whether SHP overexpression in macrophages could reverse the observed effects in *Shp*-MKO BMDMs, we utilized lentiviral transduction to introduce a Flag-SHP fusion protein into the murine macrophage cell line RAW 264.7. Stable SHP overexpression cells (PMSCV-SHP) were then selected using puromycin, while cells infected with the lentiviral vector PMSCV served as a control. The successful overexpression of SHP in PMSCV-SHP cells was confirmed through Western blot analysis (Figure 2C). Considering that Pparg acts as a master regulator of anti-inflammatory macrophage differentiation [33,34], and our previous study revealed a close relationship between Shp and Pparg, with *Shp* deletion decreasing *Pparg* mRNA expression [29], we hypothesized that SHP overexpression would increase *Pparg* expression. Given that SHP inhibits the expression of *miR-34a* [35] and *miR-34a* can target the 3′-untranslated region (3′UTR) of *Pparg* mRNA [36] to decrease *Pparg* mRNA expression [37], we further speculated that overexpression of SHP would result in decreased *miR-34a* expression and increased *Pparg* expression. To test this hypothesis, we assessed the expression levels of *miR-34a* and *Pparg* in PMSCV-SHP RAW cells and vector control cells. As expected, PMSCV-SHP cells exhibited a downregulation of *miR-34a* and upregulation of *Pparg* mRNA compared to vector control cells (Figure 2C). This indicates that SHP overexpression indeed increases the expression of *Pparg*, which should theoretically inhibit M1 macrophage differentiation. Indeed, the anticipated effect of SHP overexpression was observed, as it significantly inhibited the expression of *Nos2*, a marker of M1 proinflammatory macrophages, both in the control condition and after IFN-gamma stimulation (Figure 2D). Furthermore, we noticed a decrease in *Pparg* mRNA after IFN-gamma treatment in both PMSCV-SHP RAW cells and vector controls; however, the PMSCV-SHP RAW cells exhibited persistently higher expression of *Pparg* mRNA compared to vector control cells (Figure 2D). These results demonstrate that SHP plays a critical role as a regulator of macrophage polarization, with Pparg likely involved in the underlying mechanism. Overall, our findings emphasize the importance of SHP in modulating macrophage differentiation in vitro, as its absence promotes M1 polarization and impairs M2 polarization. Conversely, SHP overexpression increases *Pparg* mRNA expression and inhibits M1 macrophage differentiation, highlighting its potential as an important regulator of macrophage differentiation during the innate immune response.

### 3.4. Shp Knockout Leads to a Persistent Hepatic Infiltration of Proinflammatory Monocytes and M1 Macrophage Differentiation following LPS Challenge

**Figure 3:**
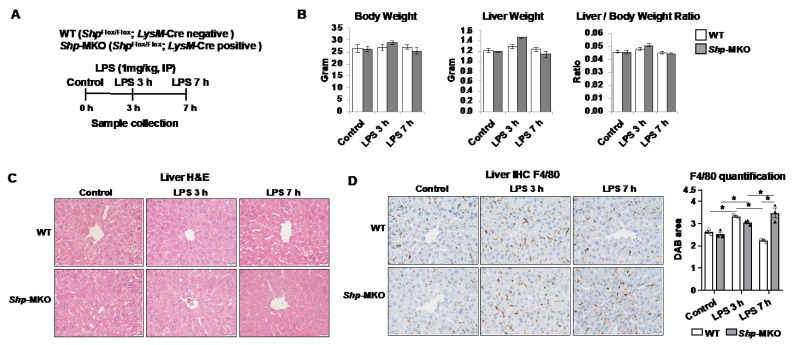
*Shp* knockout results in a persistent hepatic accumulation of macrophages following LPS challenge. (**A**) *Shp* myeloid cell specific knockout (*Shp*-MKO) was generated by breeding *Shp*^flox/flox^ with *LysM*-Cre mice. Both WT and *Shp*-MKO mice were subjected to intraperitoneal LPS (1 mg/kg body weight) injection, and samples were collected at 0-, 3-, and 7 h post-injection. (**B**) Mouse body weight, liver weight, and liver-to-body weight ratio. (**C**) Liver sections were stained with hematoxylin and eosin (H&E) to examine the histological changes in the liver. (**D**) Left, representative images of liver sections stained with macrophage marker F4/80. Original magnification, X40. Right, quantification of the DAB-positive staining area. *n* = 3/group. The data are presented as mean ± SEM for 3 samples/group. * *p* < 0.05 between the indicated groups.

To assess the impact of abnormal macrophage polarization resulting from *Shp* deletion on the immune response to endotoxin challenge, we conducted an intraperitoneal injection of a low dose of LPS (1 mg/kg body weight) in both WT and *Shp*-MKO mice. Samples were collected at 0, 3, and 7 h intervals after injection to determine the extent of the immune response (Figure 3A). Notably, the low dose of LPS did not cause significant changes in mouse body weights, liver weights, or liver-to-body weight ratios between the WT and *Shp*-MKO mice (Figure 3B). Histological examination of liver sections using H&E staining did not reveal any evident differences between the two groups after LPS injection (Figure 3C). However, immunohistochemical staining for adhesion G protein-coupled receptor E1 (Emr1 or F4/80), a surface marker of macrophages, indicated a significant increase in macrophage numbers in both WT and *Shp*-MKO livers after 3 h of LPS injection (Figure 3D). Subsequently, macrophage numbers in WT livers returned to basal levels after 7 h of LPS challenge. In contrast, the *Shp*-MKO livers maintained elevated macrophage numbers at the 7 h timepoint following LPS injection (Figure 3D). These findings suggest that myeloid *Shp* knockout mice sustain a proinflammatory signal after LPS challenge and lack an anti-inflammatory mechanism to halt macrophage accumulation during the resolution phase of inflammation.

**Figure 4:**
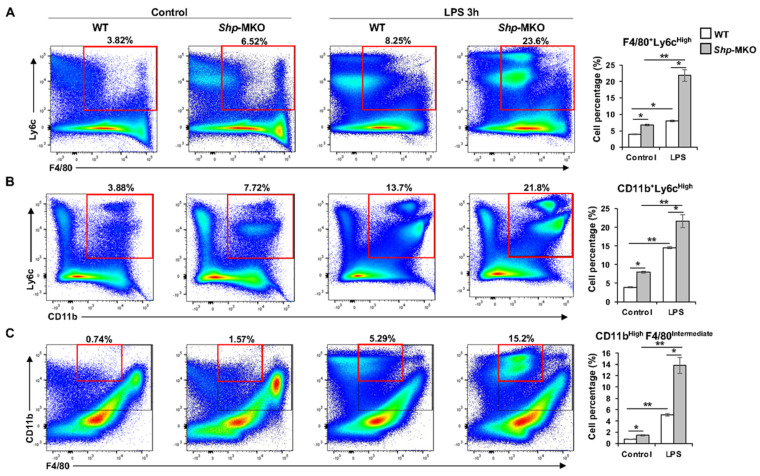
Flow cytometry analysis of composition of hepatic macrophages and monocytes following LPS challenge. WT and *Shp*-MKO mice were intraperitoneally injected with or without LPS (1 mg/kg body weight). After 3 h, mouse livers were perfused and digested to isolate nonparenchymal cells (NPCs). Approximately 1 × 10^6^ NPCs were labeled with specific antibodies and prepared for flow cytometry analysis. Single cells were gated based on FSC–A and FSC–H to exclude doublets. Dead cells stained with Zombie Aqua were excluded from the analysis. Live cells positive for CD45 expression were gated, and the populations of interest were calculated, including F4/80^+^Ly6C^High^ proinflammatory M1 macrophages (**A**), CD11b^+^Ly6C^High^ proinflammatory monocytes (**B**), and CD11b^High^F4/80^Intermediate^ monocyte-derived macrophages (**C**). The data are presented as mean ± SEM for 3 samples/group. * *p* < 0.05 and ** *p* < 0.01 between the indicated groups.

To further investigate the inflammatory phenotype and origin of hepatic macrophages, flow cytometry analysis was performed. Our hypothesis centered on the idea that the lack of *Shp* in myeloid cells would result in enhanced differentiation of M1 proinflammatory macrophages within the liver. This effect was likely attributable to the enhanced infiltration of proinflammatory monocytes into the hepatic tissue due to loss of *Shp*, particularly following the LPS challenge. To test this hypothesis, LPS (1 mg/kg body weight) was administered intraperitoneally to both WT and *Shp*-MKO mice. After 3 h, liver perfusion was performed, followed by the isolation of liver nonparenchymal cells (NPCs). Those NPCs were then subjected to staining with specific cell markers, including hematopoietic cell marker protein tyrosine phosphatase receptor type C (PTPRC or CD45), macrophage marker F4/80, monocyte marker integrin alpha M (Itgam or CD11b), and M1 proinflammatory cell marker Ly6-C antigen (Ly6C). Subsequently, the leukocyte population was isolated based on forward scatter (FSC) vs. side scatter (SSC) and then gated for CD45 expression. As anticipated, the population of proinflammatory M1-like macrophages, identified as F4/80^+^Ly6C^High^ by flow cytometry, displayed a significant increase in *Shp*-MKO livers compared to WT controls, both under basal and LPS challenge conditions (Figure 4A). Similarly, the population of proinflammatory CD11b^+^Ly6C^High^ monocytes was significantly higher in *Shp*-MKO livers compared to WT controls (Figure 4B). Additionally, under basal conditions, the population of monocyte-derived macrophages (MDMs) within the liver, identified as CD11b^High^F4/80^Intermediate^, was significantly elevated in *Shp*-MKO livers compared to WT livers (Figure 4C). Furthermore, the LPS challenge induced a substantial increase in MDM infiltration into the liver, which was nearly tripled in *Shp*-MKO livers (Figure 4C). These findings strongly suggest that the targeted deletion of *Shp* in myeloid cells fosters the infiltration of proinflammatory monocytes into the liver and enhances proinflammatory M1 macrophage differentiation in response to endotoxin.

### 3.5. Shp Deletion in Myeloid Cells Leads to Increased Cytokine Production in Response to LPS Challenge

**Figure 5:**
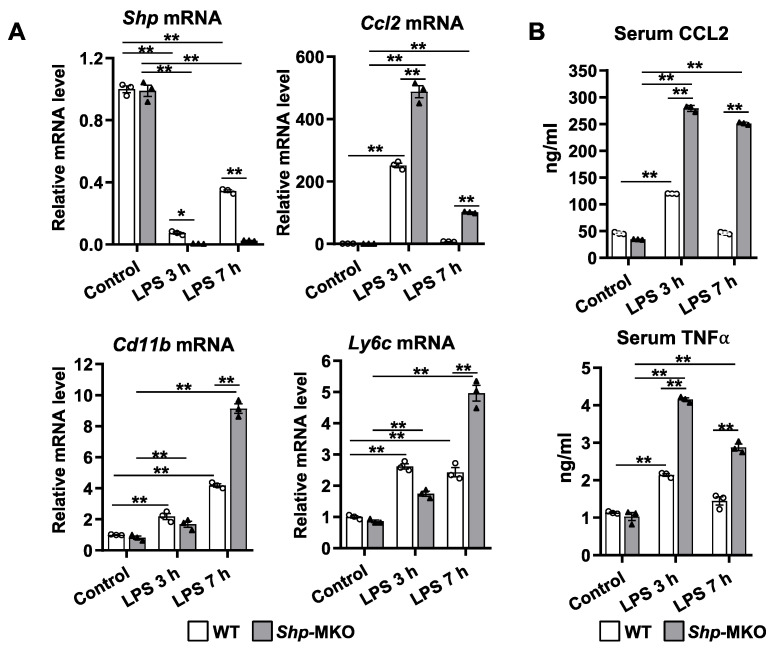
Myeloid cell specific deletion of *Shp* leads to enhanced chemokine production in response to LPS challenge. WT and *Shp*-MKO mice were intraperitoneally injected with LPS (1 mg/kg body weight), and samples were collected at 0, 3, and 7 h post-injection. (**A**) The mRNA levels of *Shp*, *Ccl2*, *Cd11b*, and *Ly6c* in liver tissues were quantified using qPCR. (**B**) The serum levels of CCL2 and TNFα were measured using ELISA to evaluate the circulating levels of these chemokines. The data are presented as mean ± SEM for 3 samples/group. * *p* < 0.05 and ** *p* < 0.01 between the indicated groups.

Our previous study has shown that LPS treatment decreased *Shp* mRNA expression in hepatocytes [28]. Consistent with our previous findings, we observed a sharp reduction in hepatic *Shp* mRNA levels at both 3 h and 7 h time points following LPS challenge in WT mice (Figure 5A). Notably, there was a partial recovery of *Shp* mRNA expression in WT livers at the 7 h time point. In contrast, *Shp* mRNA was nearly undetectable in *Shp*-MKO liver after LPS challenge at both time points (Figure 5A). LPS challenge significantly increased the mRNA levels of proinflammatory genes *Ccl2*, *Cd11b*, and *Ly6C* in WT livers, and this response was further augmented in *Shp*-MKO livers (Figure 5A). Importantly, we observed a sustained elevation in *Cd11b* and *Ly6C* mRNA levels in the *Shp*-MKO liver after 7 h of LPS challenge (Figure 5A). This finding aligns with the increased presence of proinflammatory MDMs and M1 macrophages in the *Shp*-MKO liver after LPS challenge, as detected through flow cytometry analysis (Figure 4). Consistent with the changes in *Ccl2* mRNA levels observed in WT livers, the serum concentration of CCL2 increased after 3 h of LPS challenge and returned to basal levels after 7 h (Figure 5B). A similar pattern was observed for serum TNFα levels in WT mice. Strikingly, *Shp*-MKO mice exhibited approximately 2–3 times higher induction of serum CCL2 and TNFα compared to WT controls at all time points after LPS challenge (Figure 5B). These results collectively suggest that the loss of macrophage *Shp* leads to the absence of an anti-inflammatory mechanism in *Shp*-MKO mice, highlighting the important regulatory role of myeloid *Shp* in controlling inflammatory responses.

### 3.6. Shp Deletion Results in Hyperactivation of Both MAPK and NF-κB Signaling Pathways

**Figure 6:**
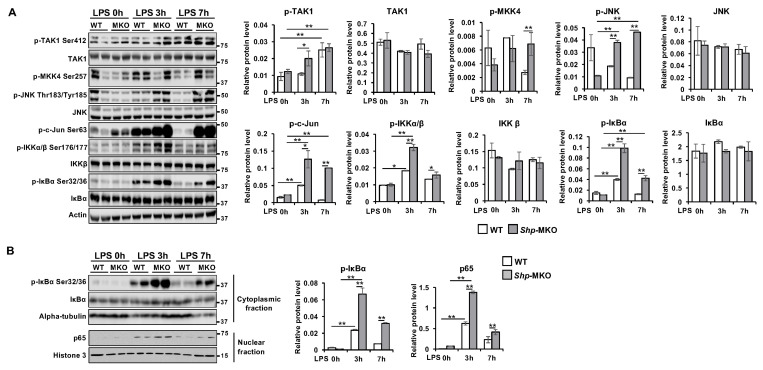
Myeloid cell specific deletion of *Shp* results in hyperactivation of MAPK and NF-κB pathways in response to LPS challenge. WT and *Shp*-MKO mice were intraperitoneally injected with LPS (1 mg/kg body weight), and samples were collected at 0-, 3-, and 7 h post-injection. (**A**) Left, Western blot analysis of whole protein lysates from liver tissues revealed the expression and phosphorylation levels of proteins involved in MAPK and NF-κB signaling pathways. Right, the protein band density was quantified using Image Studio 5.2 software, and the relative expression levels were normalized to the loading control β-actin. (**B**) Left, Western blot analysis of cytoplasmic and nuclear fractions demonstrated the expression and phosphorylation levels of proteins involved in NF-κB signaling. Right, the protein band density was quantified using Image Studio software, and the relative levels of proteins were normalized to the nuclear loading control Histone H3 and the cytoplasmic loading control α-tubulin, respectively. The data are presented as mean ± SEM. Western blots were repeated 3 times and one represented image was included in the figure. * *p* < 0.05 and ** *p* < 0.01 between the indicated groups.

Both the MAPK and NF-κB signaling pathways are well established as promoters of proinflammatory M1 macrophage differentiation [38,39,40]. To investigate whether the abnormal macrophage polarization observed in *Shp*-MKO mice could be attributed to the hyperactivation of MAPK and NF-κB signaling, we examined the activation status of several intermediate and effector proteins in these pathways in the livers of WT and *Shp*-MKO mice following LPS injection. After 3 h of LPS challenge, we observed significantly higher levels of phosphorylated proteins associated with MAPK signaling, including p-TAK1 Ser412, p-JNK Thr183/Tyr185, and p-c-Jun Ser63, in the *Shp*-MKO livers compared to WT controls (Figure 6A). Furthermore, after 7 h of LPS challenge, we observed continuous high levels of p-MKK4 Ser257, p-JNK Thr183/Tyr185, and p-c-Jun Ser63 in the *Shp*-MKO liver (Figure 6A). These findings indicate that *Shp*-MKO mice exhibit augmented MAPK activation following LPS challenge, sustaining an overall higher amplification of the immune response in *Shp*-MKO mice.

Additionally, we found that the phosphorylated proteins associated with NF-κB signaling, including p-IKKα/β Ser176/177 and p-IκBα Ser32/36, were significantly higher in the *Shp*-MKO livers compared to WT livers after both the 3 h and 7 h LPS challenges (Figure 6A). This suggests a hyperactivation of the NF-κB signaling pathway in the *Shp*-MKO liver compared to WT liver following LPS injection. To further confirm this observation, we assessed the nuclear translocation of p65, a marker of NF-κB pathway activation, and found significantly higher translocations at both the 3 h and 7 h timepoints after LPS challenge in the *Shp*-MKO liver compared to WT controls (Figure 6B). Collectively, these observations indicate that myeloid SHP negatively regulates the activation of both the MAPK and NF-κB pathways following LPS challenge.

**Figure 7:**
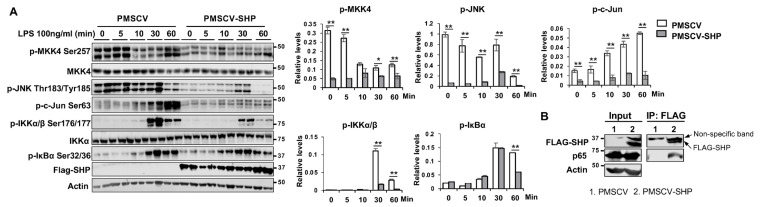
SHP overexpression hinders the activation of MAPK and NF-κB pathways in response to LPS challenge. (**A**) Left, mouse macrophage RAW cells with or without SHP overexpression were treated with 100 ng/mL LPS for different durations (0, 5, 10, 30, and 60 min). Whole cell lysates were collected and subjected to Western blot analysis to assess the expression and phosphorylation levels of proteins involved in MAPK and NF-κB signaling pathways. Right, the protein band density was quantified using Image Studio software, and the relative expression levels were normalized to the loading control β-actin. (**B**) Co-immunoprecipitation experiments were conducted using whole protein lysates from RAW cells with or without FLAG-SHP overexpression. The protein–protein interaction of SHP with p65 was detected by Western blot analysis. The data are presented as mean ± SEM. Western blots were repeated 3 times and one represented image was included in the figure. * *p* < 0.05 and ** *p* < 0.01 between the indicated groups.

To gain deeper insights into the role of SHP in regulating these pathways, we conducted experiments using RAW cells overexpressing SHP (PMSCV-SHP) and vector control PMSCV RAW cells. These cells were treated with LPS (100 ng/mL) for varying durations (0, 5, 10, 30, and 60 min), and we examined the expression of proteins involved in the MAPK and NF-κB pathways. Our results demonstrated that LPS treatment led to time-dependent changes in the expression of p-MKK4 Ser257, p-JNK Thr183/Tyr185, p-c-Jun Ser63, p-IKKα/β Ser176/177, and p-IκBα Ser32/36 (Figure 7A). Remarkably, the overexpression of SHP in macrophages resulted in a reduction in these phosphorylated proteins after LPS treatment (Figure 7A). Prior studies have highlighted SHP’s ability to negatively regulate the function of various transcription factors and nuclear receptors through direct protein–protein interactions [16]. Building on this knowledge, we delved further into the potential interactions between SHP and proteins in the MAPK and NF-κB pathways. We employed anti-FLAG magnetic beads to selectively capture the FLAG-SHP fusion protein, which was deliberately overexpressed in the protein lysates obtained from the PMSCV-SHP cells. Notably, the subsequent pulldown analysis revealed the presence of p65 exclusively in PMSCV-SHP cell samples, while it remained absent in PMSCV control cells (Figure 7B). This conspicuous distinction strongly indicates the existence of a protein–protein interaction between SHP and p65. This pivotal observation finds congruence with the previously published studies from our own laboratory and other groups [26,28]. Collectively, these findings conclusively establish that SHP plays a pivotal role in regulating immune response by inhibiting both MAPK and NF-κB signaling pathways.

## 4. Discussion

Hepatic macrophages are key players in innate immunity and vital components of the liver. These macrophages display remarkable heterogeneity and plasticity, allowing them to respond to diverse stimuli in different physiological and pathological conditions [41,42,43,44]. Traditionally, macrophages have been classified into two extreme groups: M1, classically activated proinflammatory macrophages; and M2, alternatively activated anti-inflammatory macrophages. However, emerging research employing single-cell RNA sequencing has unveiled the intricate nature of macrophage differentiation, showcasing a multitude of activation states that surpass the conventional M1/M2 classification. Nevertheless, despite this newfound complexity, the M1/M2 classification continues to serve as a valuable framework for understanding macrophage function and gene roles, offering a useful overview in the study of macrophage biology. SHP is an atypical nuclear receptor that plays a critical role in various pathophysiological processes, including inflammation, metabolism, and energy homeostasis [17,19,26]. Our previous research has highlighted the anti-inflammatory role of hepatic SHP in a mouse model of nonalcoholic steatohepatitis [28,29]. Building upon these findings, our current study aimed to investigate the role of myeloid SHP in macrophage polarization during acute innate immune response. We discovered that SHP regulates macrophage polarization, and its influence on M1 and M2 differentiation is an important mechanism through which SHP inhibits inflammation. Mechanistically, we made the novel discovery that myeloid SHP modulates macrophage differentiation by regulating the Pparg, MAPK, and NF-κB pathways.

An intriguing finding of our study is the alteration of SHP expression during macrophage polarization. Specifically, anti-inflammatory M2 macrophage differentiation led to an increase in *Shp* mRNA expression, while proinflammatory M1 macrophage differentiation resulted in its decrease. Although the precise mechanisms underlying this regulation are unknown and beyond the scope of our current study, previous studies have demonstrated that several nuclear receptors and transcription factors bind to the *Shp* gene promoter and influence its expression [16]. For instance, PPARg binds to the PPAR response element on the *Shp* gene promoter and induces *Shp* mRNA expression [45]. Macrophage-stimulating factor (MSP) increases *Shp* mRNA expression through the activation of AMP-activated protein kinase (AMPK) pathway [46,47]. Considering that PPARg and AMPK pathways are upregulated during M2 macrophage differentiation [48,49], it is tempting to speculate that the increase in *Shp* mRNA in M2 macrophages may be attributed to the activation of these pathways. Conversely, JNK activation suppresses *Shp* transcription in hepatocytes [28], and JNK activation is required for M1 macrophage polarization [50]. Hence, it is possible that JNK activation leads to the decreased *Shp* mRNA expression in M1 macrophages. Further investigations are warranted to explore whether manipulating PPARg, AMPK, or JNK in macrophages can alter SHP expression during macrophage differentiation.

Motivated by the differential SHP expression observed in M1 and M2 macrophages, we sought to determine whether SHP plays a functional role in macrophage polarization. To this end, we generated a genetic mouse model lacking *Shp* specifically in myeloid cells using LysM-Cre-mediated knockout. We isolated BMDMs from both *Shp*-MKO and WT controls and found that *Shp* loss inhibited the polarization of BMDMs toward an M2 phenotype while promoting polarization toward an M1 state. Moreover, overexpression of SHP in the macrophage cell line RAW cells decreased miR-34a expression but increased *Pparg* mRNA expression and inhibited macrophage polarization toward a M1 phenotype. Encouraged by these in vitro results, we conducted in vivo studies by injecting a low dose of LPS into *Shp*-MKO and WT controls. We observed a sustained increase in liver macrophage numbers in *Shp*-MKO mice following LPS challenge. Notably, flow cytometry revealed a higher population of CD11b^High^ F4/80^Intermediate^ monocyte-derived macrophages in *Shp*-MKO livers compared to WT controls after LPS challenge, suggesting that the loss of SHP in myeloid cells enhances monocyte infiltration into the liver, replenishing hepatic macrophage populations. Monocyte recruitment to the liver is finely regulated by chemokines, among which CCL2 plays a crucial role. Inhibition of CCL2 or genetic knockout of *Ccl2* specifically in myeloid cells has been shown to reduce monocyte infiltration into the liver during both acute and chronic hepatic injury [51,52,53]. In our previous study, we found that the loss of *Shp* in hepatocytes triggers the production of CCL2, leading to the initiation of monocyte recruitment [28]. Hence, we postulated that the increased monocyte infiltration observed in *Shp*-MKO livers after LPS challenge might be due to elevated CCL2 levels following SHP loss in myeloid cells. Our observations confirmed this speculation, as we noted a significant increase in hepatic *Ccl2* mRNA expression and elevated serum CCL2 levels in *Shp*-MKO mice. These results suggest that SHP universally regulates CCL2 expression in various cell types, contributing to the enhanced monocyte infiltration observed in *Shp*-MKO livers during the response to LPS challenge.

Our flow cytometry analysis yielded a significant discovery, revealing increased populations of F4/80^+^Ly6C^High^ and CD11b^+^Ly6C^High^ cells in *Shp*-MKO livers compared to WT controls. Ly6C, a member of the lymphocyte antigen-6 (Ly6)/urokinase-type plasminogen activator receptor protein superfamily, is closely associated with infiltrating monocytes and is involved in the production of proinflammatory cytokines and chemokines, such as interleukin 1 (IL-1), interleukin 18 (IL-18), and Ccl2 [54]. Ly6C^High^ monocytes are recognized as a proinflammatory subset contributing to tissue inflammation and T-cell activation [55]. Notably, Ly6C^High^ monocytes’ infiltration and their subsequent differentiation into proinflammatory M1 macrophages are considered crucial early steps in liver inflammation [53]. In our study, flow cytometry analysis demonstrated a notable increase in proinflammatory CD11b^+^Ly6C^High^ monocytes and F4/80^+^Ly6C^High^ M1-like macrophages in *Shp*-MKO livers compared to WT controls, both under basal conditions and after LPS challenge. These findings suggest that the loss of *Shp* in myeloid cells promotes the infiltration of proinflammatory monocytes into the liver and enhances their differentiation into proinflammatory M1 macrophages. The precise mechanisms by which SHP regulates the expression of Ly6C remain unclear. Additional studies are needed to unravel the molecular pathways and signaling events through which SHP modulates Ly6C expression in myeloid cells.

Pparg, MAPK, and NF-κB are pivotal regulators in the intricate regulation of macrophage activation and differentiation [39,56]. In our study, we observed that mice lacking myeloid *Shp* exhibited a pronounced activation of the MAPK and NF-κB pathways in the liver following LPS challenge. In contrast, when SHP was overexpressed in macrophages, it triggered an increase in *Pparg* mRNA expression while concurrently inhibiting the activation of both MAPK and NF-κB signaling. Additionally, our study unearthed a noteworthy finding: a direct protein–protein interaction between SHP and p65. This discovery aligns with earlier research, which illuminated SHP’s multifaceted role within the NF-κB signaling pathway, portraying it not only as a repressor of p65 subunit transactivation but also as a potent inhibitor of TRAF6 adaptor-mediated polyubiquitination [26]. SHP achieves this through direct interactions with p65 and TRAF6. Notably, TRAF6 is responsible for catalyzing the attachment of Lys63 (K63)-linked polyubiquitin chains to NF-κB modulator IKKγ [57,58]. Consequently, the ubiquitination of IKKγ activates the IKK complex IKKα/β in the *Shp*^−/−^ macrophages, resulting in increased phosphorylation of IκBα protein and augmented nuclear translocation of p65.

Our study provides the first evidence of SHP’s regulatory role in impeding MAPK activation. To delve into the mechanistic underpinning of SHP’s influence on MAPK pathways, our initial hypothesis centered on the possibility of SHP engaging with key modulators within the MAPK cascade to exert inhibitory effects. However, our immunoprecipitation experiment failed to unveil any discernible protein–protein interactions between SHP and MAPK proteins. As a result, it seems improbable that SHP’s modulation of MAPK pathways occurs via direct protein interactions. Nonetheless, it is pertinent to note that TRAF6, a pivotal adaptor molecule in the TLR signaling cascade, also occupies a crucial role in the activation of MAPK pathways [59]. Stimulation of macrophages with LPS is known to trigger TRAF6-dependent activation of the MAPK pathway [60]. Thus, the heightened MAPK pathway activation observed in *Shp*^−/−^ macrophages could potentially stem from an augmented TRAF6 activity, offering a plausible explanation for the observed outcomes in current study.

One limitation of our study is the use of the LyzCre system, which achieves high-level gene knockout in myeloid cells, including monocytes and macrophages [61]. However, the LyzCre knockout strategy also affects a subset of neutrophils [62]. Therefore, some of the proinflammatory phenotypes observed in the *Shp*-MKO mice, such as increased cytokine and chemokine production, may also be attributed to *Shp* loss in neutrophils. Future studies employing alternative conditional macrophage-specific gene knockout models will be necessary to confirm our results obtained from the LyzCre system.

## 5. Conclusions

**Figure 8:**
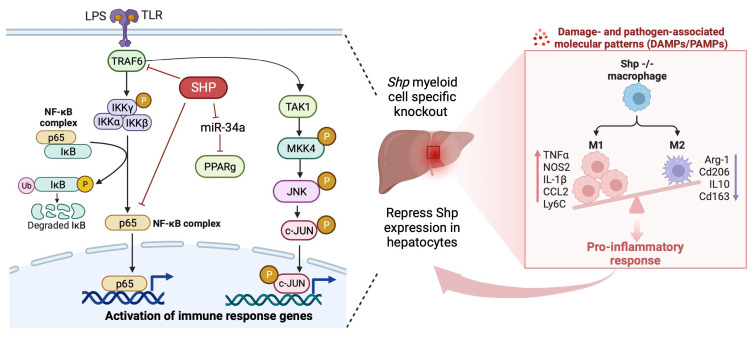
Schematic diagram illustrating the crucial role of SHP in regulating macrophage polarization and its significant impact on the immune response during LPS-induced inflammation. The absence of *Shp* in myeloid cells results in the augmented infiltration of proinflammatory monocytes and their subsequent differentiation into proinflammatory M1 macrophages upon LPS challenge. These effects are attributed to the dysregulation of Pparg, MAPK, and NF-κB signaling pathways due to the loss of *Shp* in macrophages, further contributing to the persistent accumulation of proinflammatory M1 macrophages and the downregulation of hepatic *Shp* expression through the interactions between monocytes/macrophages and hepatocytes.

Our study has unveiled that the absence of *Shp* in myeloid cells results in an augmented infiltration of proinflammatory monocytes and their subsequent differentiation into proinflammatory M1 macrophages upon LPS challenge. These effects can be attributed to the dysregulation of Pparg, MAPK, and NF-κB signaling pathways due to the loss of *Shp* in macrophages, further contributing to the persistent accumulation of proinflammatory M1 macrophages and the downregulation of hepatic *Shp* expression through the interactions between monocytes/macrophages and hepatocytes (Figure 8). This sustained accumulation of proinflammatory macrophages in the liver highlights the crucial role of SHP in regulating macrophage polarization and its significant impact on the immune response during LPS-induced inflammation. Overall, our findings shed light on the intricate mechanisms through which SHP influences macrophage behavior, thereby significantly contributing to our understanding of the complex interplay between SHP, macrophage polarization, and the innate immune response. This research enhances our comprehension of the underlying processes involved in immune regulation and may have implications in the development of novel therapeutic approaches for inflammatory conditions.

## Data Availability

All data analyzed during this study are included in this published article.

## Supplementary Materials

The following supporting information can be downloaded at: https://www.mdpi.com/article/10.3390/biomedicines11092403/s1, Table S1: Primers for qPCR.

## Abbreviations

SHP, small heterodimer partner; Pparg, peroxisome proliferator activated receptor gamma; LPS, lipopolysaccharide; MDM, monocyte-derived macrophages; BMDM, bone marrow-derived macrophages; *Shp*-MKO, *Shp* myeloid cell specific knockout; *Tnfa*, tumor necrosis factor alpha; Ccl2, chemokine (C-C motif) ligand 2; MAPK, mitogen-activated protein kinase; NF-κB, nuclear factor kappa B; KC, Kupffer cells; IFN-gamma, interferon gamma; IL4, interleukin 4; NASH, nonalcoholic steatohepatitis; Nos2, nitric oxide synthase 2; Arg1, arginase 1; Cd163, CD163 antigen; Mrc1, mannose receptor C type 1; 3′UTR, 3′-untranslated region; Emr1 or F4/80, adhesion G protein-coupled receptor E1; NPCs, nonparenchymal cells; PTPRC or CD45, protein tyrosine phosphatase receptor type C; Itgam or CD11b, monocyte marker integrin alpha M; Ly6C, Ly6-C antigen; FSC, forward scatter; SSC, side scatter; Co-IP, co-immunoprecipitation; MSP, macrophage-stimulating factor; AMPK, AMP-activated protein kinase; IL-1, interleukin 1; IL-18, interleukin 18.
