# Supplementary material for: Small Heterodimer Partner Modulates Macrophage Differentiation during Innate Immune Response through the Regulation of Peroxisome Proliferator Activated Receptor Gamma, Mitogen-Activated Protein Kinase, and Nuclear Factor Kappa B Pathways
Source: Biomedicines. 2023 Aug 28;11(9):2403. doi: 10.3390/biomedicines11092403 (PMC10525324; doi:10.3390/biomedicines11092403)
Supplement: Supplementary file 1 [file biomedicines-11-02403-s001.zip › biomedicines-2549511-supplementary.pdf]

Table S1. **Primers for qPCR**

Gene name	Sequence
Mouse *Shp*	F: 5'-TGAGCTGGGTCCCAAGGA-3'<br>R: 5'-CCTGGCACATCTGGGTTGA-3'
Mouse *Actin*	F: 5'- GGCTGTATTCCCCTCCATCG-3'<br>R: 5'- CCAGTTGGTAACAATGCCATGT-3'
Mouse *TNFα*	F: 5'- CGTGGAACTGGCAGAAGAG -3'<br>R: 5'- ACAAGCAGGAATGAGAAGAGG -3'
Mouse *Ccl2*	F: 5'- TTAAAAACCTGGATCGGAACCAA -3'<br>R: 5'- GCATTAGCTTCAGATTTACGGGT-3'
Mouse *Nos2*	F: 5'- AATCTTGGAGCGAGTTGTGG -3'<br>R: 5'- CAGGAAGTAGGTGAGGGCTTG -3'
Mouse *Arg1*	F: 5'- CTCCAAGCCAAAGTCCTTAGAG -3'<br>R: 5'- AGGAGCTGTCATTAGGGACATC -3'
Mouse *CD163*	F: 5'- TGGGTGGGGAAAGCATAACT -3'<br>R: 5'- AAGTTGTCGTCACACACCGT -3'
Mouse *CD206*	F: 5'- CTCTGTTCAGCTATTGGACGC -3'<br>R: 5'- CGGAATTTCTGGGATTCAGCTTC -3'
Mouse *CD11b*	F: 5'- ATGGACGCTGATGGCAATACC -3'<br>R: 5'- TCCCCATTCACGTCTCCCA -3'
Mouse *Ly6C*	F: 5'- GCAGTGCTACGAGTGCTATGG -3'<br>R: 5'- ACTGACGGGTCTTTAGTTTCCTT -3'
